# Effects of Pandemics-Related Uncertainty on Household Consumption: Evidence From the Cross-Country Data

**DOI:** 10.3389/fpubh.2020.615344

**Published:** 2020-11-24

**Authors:** Shuiting Wu

**Affiliations:** Guangxi University of Finance and Economics, Nanning, China

**Keywords:** household consumption, pandemics-related uncertainty, world pandemic uncertainty index, COVID-19 related uncertainty, panel data estimations

## Abstract

The COVID-19 pandemic has affected various macroeconomic indicators. Given this backdrop, this research investigates the effects of the pandemics-related uncertainty on household consumption. For this purpose, we construct a simple theoretical model to study the effects of the pandemics-related uncertainty on household consumption. To estimate the theoretical model, we consider the panel dataset of 138 countries for the period from 1996 to 2017. We also use the Pandemic Uncertainty Index to measure the pandemics-related uncertainty. The theoretical model and the empirical findings from the Feasible Generalized Least Squares (FGLS) estimations indicate that the gross fixed capital formation, government consumption, balance of trade, and the Pandemic Uncertainty Index negatively affect household consumption. The results are also valid in the panel dataset of 42 high-income economies and the remaining 96 emerging economies.

## Introduction

COVID-19 Pandemic has negatively affected every aspect of the world economy. Since new coronavirus is more deadly than virus related to regular flu, governments have implemented various policy implications to slow down the spread of the virus. Specifically, policymakers have closed downed the public areas, including schools, restaurants, shopping malls (1) or people have voluntarily stayed at home during the COVID-19 Pandemic (2).

Although the COVID-19 Pandemic has one of the most unprecedented pandemics in the modern history, there are several pandemics in the twenty-first century, such as the Severe Acute Respiratory Syndrome (SARS) (2002**–**2003), Avian Flu (2003**–**2009), Swine Flu (2009**–**2010), Bird Flu (2013**–**2017), Ebola (2014**–**2016), and Middle East Respiratory Syndrome (MERS) (2014**–**Ongoing). It is observed that most of these pandemics have spread out at the regional level, or they have limited effects on economic indicators (3). However, these pandemics show us how the COVID-19 Pandemic can affect the macroeconomic indicators. In this paper, we aim to examine the effects of the pandemics-related uncertainty on the household consumption.

According to Altig et al. (4), economic uncertainty in the global economy during the COVID-19 Pandemic is higher than the level before the COVID-19 Pandemic. Baker et al. (5) show that the COVID-19 Pandemic related economic uncertainty has significantly macroeconomic indicators (consumption, employment, and investments) as well as it is negatively related to the stock market returns. Leduc and Liu (6) also indicate that the COVID-19 related uncertainty is the significant driver of the macroeconomic indicators. Following these papers, we focus on the Pandemic Uncertainty Index of Ahir et al. (3) to measure the pandemics-related uncertainty.

There are previous papers to investigate the effects of economic uncertainty related to the COVID-19 Pandemic on household consumption. For instance, Baker et al. (7) observe that people in the United States increased total spending by over 40% during the early March 2020, but household consumption has been reduced by around 30% during the late March 2020. The authors also show that food delivery and grocery spending are major exceptions to this reduction. Using the bank card and mobile Quick Response (QR) code transactions data, Chen et al. (8) also show that the household spending in China has significantly declined during the late January 2020. Consumption of goods and services has significantly decreased by 33 and 34%, respectively. The authors estimate that the decline of the household consumption in 2020Q1 is around 1.2% of China's GDP in 2019. Finally, Martin et al. (9) use the San Francisco Bay Area as a case study of the lockdown implications. The authors consider the household-level data to examine the effects of the COVID-19 Pandemic on the consumer spending and the poverty rate. The authors observe that there is a significant indirect macroeconomic effects of the COVID-19 Pandemic on the related variables and uncertainty related to the COVID-19 is can be defined as a typical exogenous shock, such as natural disasters. These findings motivate us to examine the effects of pandemic-related uncertainty on household consumption, but further, we aim to enhance the findings with the cross-country data.

In this paper, we construct a theoretical model to study the effects of the pandemics-related uncertainty on household consumption. To estimate our theoretical model, we consider the panel dataset of 138 countries (42 high-income economies and 96 emerging economies) for the period from 1996 to 2017. To the best of our knowledge, this is the first paper in the literature that examine the effects of the pandemics-related uncertainty on household consumption by using the cross-country data. According to the theoretical model and the empirical estimations from the FGLS method, the gross fixed capital formation, the government consumption, the balance of trade, and the Pandemic Uncertainty Index negatively affect household consumption. These results are also valid in the panel dataset of 42 high-income economies and 96 emerging economies.

The rest of the study is organized as follows. Section constructs a theoretical model to study the effects of pandemic-related uncertainty on household consumption. Section Data, empirical model and estimation procedure provides the data and the details of the model and the estimation procedure. Section discusses the empirical results. Section concludes.

## Theoretical Model

In this paper, we examine the determinants of household consumption. For this purpose, we construct a theoretical framework, which is based on the income-expenditure model in an open economy [see, e.g., (10)], and it can be written as such:

(1) $$\begin{array}{l} Y_{t}=C_{t}+I_{t}+G_{t}+\left(X_{t}-M_{t}\right) \end{array}$$

We can extract the household consumption (*C*_*t*_), and (*X*_*t*_−*M*_*t*_) can be defined as the balance of trade (*BOT*_*t*_) as follows:

(2) $$\begin{array}{l} {C_{t}=Y}_{t}-I_{t}-G_{t}-BOT_{t} \end{array}$$

At this stage, we assume that the Pandemic Uncertainty Index (PUI) captures the business cycles (economic performance) and it should be negatively related to the gross domestic product (GDP) (*Y*_*t*_) over time. Therefore, we replace *Y*_*t*_ with (−*PUI*_*t*_) and Equation (2) can be rewritten as such:

(3) $$\begin{array}{l} {C_{t}=-PUI}_{t}-I_{t}-G_{t}-BOT_{t} \end{array}$$

Note that *I*_*t*_ is also the gross fixed capital formation and *G*_*t*_ is the government consumption in this theoretical framework.

## Data, Empirical Model, and Estimation Procedure

The theoretical model in Equation (3) can be estimated via the panel data in the current form, and the estimated model can be written as follows:

(4) $$\begin{array}{l} {C_{i,t}=\alpha_{0}-\alpha_{1}PUI}_{i,t}-{\alpha_{2}I}_{i,t}-{\alpha_{3}G}_{i,t}-{\alpha_{4}BOT}_{i,t} \end{array}$$

In Equation (4), *C*_*i, t*_ is the household consumption, *PUI*_*i, t*_ is the Pandemic Uncertainty Index, *I*_*i, t*_ is the gross capital formation, *G*_*i, t*_ is the government consumption, *BOT*_*i, t*_ is the balance of trade in country *i* and time *t*.

Household consumption, gross capital formation, government consumption, and the balance of trade data are obtained from the Penn World Table (PWT) (version 9.1) dataset, which are provided by Feenstra et al. (11). All these variables are defined as the shares of the current Purchasing Power Parity (PPP) GDP.

The Pandemic Uncertainty Index (PUI) is introduced by Ahir et al. (3). This new dataset measures discussions about pandemics at the country level. The PUI is calculated by counting the number of words related to pandemics uncertainty (and its variants) in the Economist Intelligence Unit (EIU) country reports. Note that a higher level of the index indicates a greater pandemics-related uncertainty.^1^

At this stage, the empirical exercise is implemented in 138 countries for the period from 1996 to 2017. The selection of the sample is related to the data availability. Following the definition of the World Bank (12), we also split the data as 42 high-income economies^2^ and 96 emerging (low-income, lower-middle-income, and upper-middle-income) economies.^3^ Finally, a summary of descriptive statistics and the correlation matrix for variables in the estimations are provided in Table 1.

**Table 1:** Summary of descriptive statistics & correlation matrix.

**Variable**	**Mean**	**Std. Dev**.	**Min**.	**Max**.	**Obs**.
C	0.632	0.165	0.025	1.547	3,036
I	0.214	0.085	0.001	0.745	3,036
G	0.174	0.076	0.008	0.619	3,036
BOT	–0.031	0.139	−1.185	0.758	3,036
PUI	0.489	6.249	0.000	225.8	3,036
**Variable**	**C**	**I**	**G**	**BOT**	**PUI**
C	1	–	–	–	–
I	–0.5021	1	–	–	–
G	–0.2896	–0.0928	1	–	–
BOT	–0.6262	0.0434	–0.1382	1	–
PUI	–0.0241	–0.0133	0.0100	–0.0001	1

The correlation matrix indicates that there is a negative correlation between household consumption and the explanatory variables is negative. The PUI is negatively related to the gross capital formation and the balance of trade, while it is positively correlated with the government consumption.

We utilize the FGLS estimations to estimate the empirical model in Equation (4). At this point, we check the stationarity of the variables by implementing the panel unit root test of Pesaran (13).^4^ Given that the Cross-sectional Augmented Im–Pesaran–Shin (CIPS) panel unit root test of Pesaran (13) considers the cross-sectional dependence, we check the cross-sectional dependence of the variables. Therefore, we utilize the Cross-Sectional Dependence (CD) test of Pesaran (14, 15), and then we proceed with the CIPS panel unit root test of Pesaran (13). All of these results indicate that the FGLS estimations are suitable.

## Empirical Results

### Cross-Sectional Dependence (CD) Test

The results of the CD test of Pesaran (14, 15) are reported in Table 2.

**Table 2:** Cross-sectional dependence (CD) Test of Pesaran (2004 and 2015).

**Test statistics**	**C**	**I**	**G**	**BOT**	**PUI**
CD-test	14.50^***^	39.31^***^	31.58^***^	29.21^***^	282.7^***^
*P*	[0.000]	[0.000]	[0.000]	[0.000]	[0.000]
Absolute correlation	0.201	0.222	0.226	0.227	0.638

The null hypothesis of cross-section independence. The probability values in [ ]

*** *p < 0.01*.

The results in Table 2 indicate that all variables have cross-section dependence. Therefore, we proceed with a second-generation panel unit root test rather than a first-generation panel unit root test.

### CIPS Panel Unit Root Test

We also utilize the CIPS panel unit root test of Pesaran (13), and the results are provided in Table 3.

**Table 3:** Cross-sectional augmented Im–Pesaran–Shin (CIPS) test of Pesaran (13).

**Variable**	**Specification without Trend**
C	–2.879^***^ (0)
I	–6.087^***^ (0)
G	–4.995^***^ (0)
BOT	–5.324^***^ (0)
PUI	–8.479^***^ (0)

*The null hypothesis is that the series follows a unit root process. The lags are in ( )*.

*** *p < 0.01*.

The findings in Table 3 show that the null hypothesis, series follow a unit root process, are significantly rejected for all variables. Therefore, all variables in the empirical analysis are stationary, and we proceed with the FGLS estimations.

### FGLS Estimations

The results of the FGLS estimations for Equation (4) are reported in Table 4.

**Table 4:** FGLS Estimations: pandemics and household consumption (1996–2017).

**Regressors**	**(I)**	**(II)**	**(III)**
	**All countries**	**High-income economies**	**Emerging economies**
Pandemic uncertainty index_t_	−0.072^***^ (0.020)	−0.028^**^ (0.014)	−0.131^***^ (0.022)
Gross fixed capital formation_t_	−0.973^***^ (0.013)	−0.972^***^ (0.043)	−0.885^***^ (0.010)
Government consumption_t_	−0.913^***^ (0.016)	−0.664^***^ (0.033)	−0.967^***^ (0.016)
Balance of trade_t_	−0.771^***^ (0.012)	−0.781^***^ (0.016)	−0.722^***^ (0.011)
Constant term	Yes	Yes	Yes
Observation	3,016	924	2,112
Number of Countries	138	42	96
Wald Chi-square	15782.1^***^	27536.2^***^	13027.5^***^

*The dependent variable is the household consumption. Robust standard errors are in ( )*.

*** p < 0.01 and **p < 0.05.

The findings in Column (I) of Table 4 provides the findings for all (138) countries, while Columns (II) and (III) report the results for 42 high-income economies and 96 emerging economies, respectively.

The results of the FGLS estimations indicate that the PUI negatively affects the household consumption. The coefficient is −0.072 for all countries, −0.028 for the high-income economies, and it is found as −0.131 for the emerging economies, respectively. The coefficients are statistically significant at the 5% level at least. Besides, the gross capital formation, government consumption, and the balance of trade are negatively associated with the household consumption. These coefficients are also statistically significant at the 1% level. The results are valid in all countries, the high-income economies, and the emerging economies. Finally, the evidence from the FGLS estimations is in line with the expectation of the theoretical model provided in section Theoretical model.

## Conclusion

In this paper, we examined the effects of pandemics-related uncertainty on household consumption in the panel dataset of 138 countries for the period from 1996 to 2017. For this purpose, we constructed a theoretical model to study the effects of pandemics-related uncertainty on household consumption. We also use the PUI measure, which is provided by Ahir et al. (3) at https://worlduncertaintyindex.com/data/. The theoretical model and the empirical results from the FGLS estimations indicate that the gross fixed capital formation, the government consumption, the balance of trade, and the Pandemic Uncertainty Index negatively affect the household consumption. The results are also valid in the panel datasets of 42 high-income economies and 96 emerging economies.

Our findings are in line with the findings of Baker et al. (7) and Chen et al. (8). However, these papers observe that household consumption has been significantly reduced during the COVID-19 Pandemic in the United States and China, respectively. In this paper, we enhanced their findings to the panel dataset of 138 countries using the pandemic-related uncertainty before the COVID-19 period. Given that our results show that consumption is negatively related to the pandemic-related uncertainty, increasing government expenditures during the pandemics can help to sustain economic performance. Future studies can use pandemic-related uncertainty indices to examine the effects of the COVID-19 related uncertainty on other financial and macroeconomic indicators. At this juncture, time-series analyses can be implemented.

## Data Availability Statement

Publicly available datasets were analyzed in this study. This data can be found here: https://worlduncertaintyindex.com/ https://www.rug.nl/ggdc/productivity/pwt.

## Author Contributions

The author confirms being the sole contributor of this work and has approved it for publication.

## Conflict of Interest

The author declares that the research was conducted in the absence of any commercial or financial relationships that could be construed as a potential conflict of interest.

## Footnotes

^1^Refer to https://worlduncertaintyindex.com/data for further details of the dataset.

^2^Australia, Austria, Belgium, Canada, Chile, Croatia, the Czech Republic, Denmark, Finland, France, Germany, Greece, Hong Kong SAR, Hungary, Ireland, Israel, Italy, Japan, Korea Republic, Kuwait, Latvia, Lithuania, the Netherlands, New Zealand, Norway, Panama, Poland, Portugal, Qatar, Romania, Saudi Arabia, Singapore, Slovakia, Slovenia, Spain, Sweden, Switzerland, Taiwan, the United Arab Emirates, the United Kingdom, the United States, and Uruguay.

^3^Albania, Algeria, Angola, Argentina, Armenia, Azerbaijan, Bangladesh, Belarus, Benin, Bolivia, Bosnia and Herzegovina, Botswana, Brazil, Bulgaria, Burkina Faso, Burundi, Cambodia, Cameroon, Central African Republic, Chad, China, Colombia, Congo Republic, Congo DR, Costa Rica, Côte d'Ivoire, Dominican Republic, Ecuador, Egypt, El Salvador, Ethiopia, Gabon, Gambia, Georgia, Ghana, Guatemala, Guinea, Guinea-Bissau, Haiti, Honduras, India, Indonesia, Iran, Iraq, Jamaica, Jordan, Kazakhstan, Kenya, Kyrgyzstan, Laos, Lebanon, Lesotho, Liberia, Madagascar, Malawi, Malaysia, Mali, Mauritania, Mexico, Moldova, Morocco, Mozambique, Myanmar, Namibia, Nepal, Nicaragua, Niger, Nigeria, North Macedonia, Oman, Pakistan, Paraguay, Peru, the Philippines, Russian Federation, Rwanda, Senegal, Sierra Leone, South Africa, Sri Lanka, Sudan, Tajikistan, Tanzania, Thailand, Togo, Tunisia, Turkey, Turkmenistan, Uganda, Ukraine, Uzbekistan, Venezuela, Vietnam, Yemen, Zambia, and Zimbabwe.

^4^As a robustness check, we also run the Panel-Corrected Standard Errors (PCSE) estimations, and the results are in line with the FGLS estimations. We did not report them to save space due to the page constraints.
